# Supplementary material for: Antithrombotic Strategies in Patients with Atrial Fibrillation Following Percutaneous Coronary Intervention: A Systemic Review and Network Meta-Analysis of Randomized Controlled Trials
Source: J Clin Med. 2020 Apr 8;9(4):1062. doi: 10.3390/jcm9041062 (PMC7231136; doi:10.3390/jcm9041062)
Supplement: Supplementary file 1 [file jcm-09-01062-s001.pdf]

Supplementary Data

Supplementary Figure 1. Convergence of iterations was evaluated using Gelman–Rubin–Brooke statistic (Right) and trace plots (Left). All the results showing that the four Markov chain Monte Carlo (MCMC) chains mixed and converged well. (A = VKA + DAPT; B = VKA + Aspirin or P_2Y_{12} inhibitor; C = NOAC + DAPT; D = NOAC + P_2Y_{12} inhibitor)

A. TIMI Bleeding (Major)

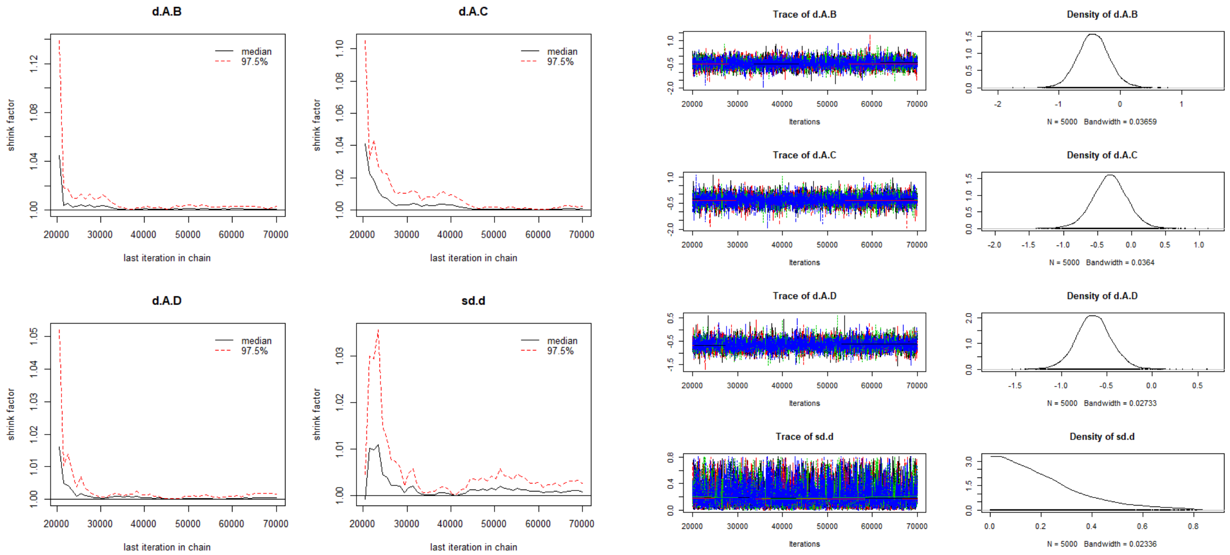

B. TIMI bleeding (Major and minor)

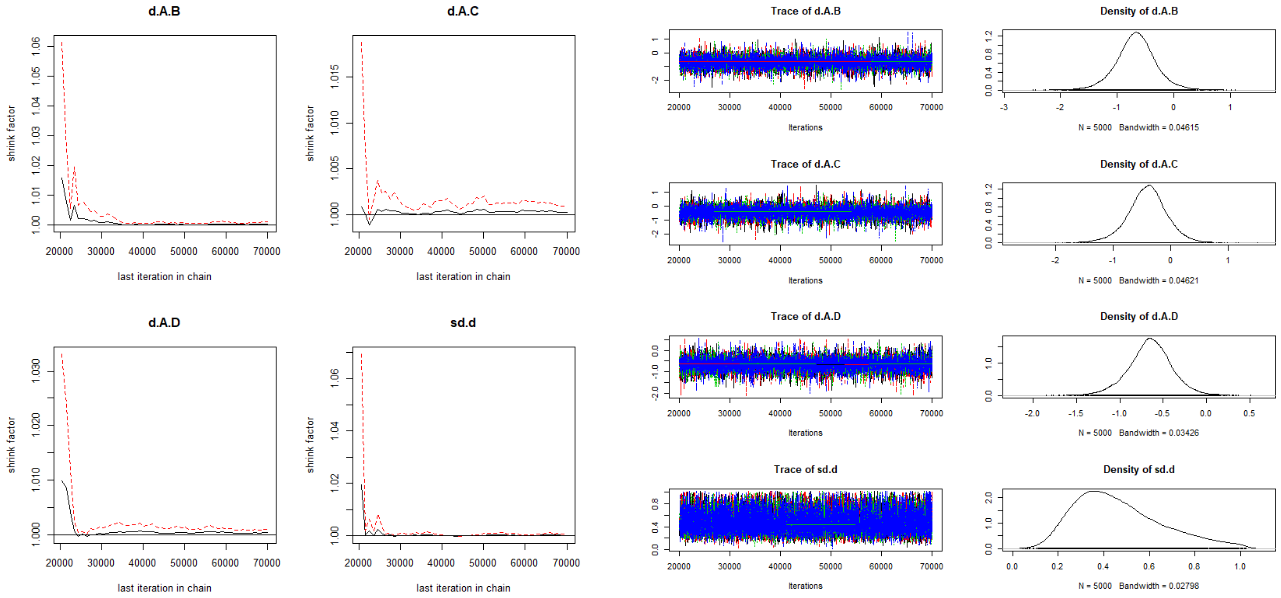

C. Trial-defined primary bleeding outcome

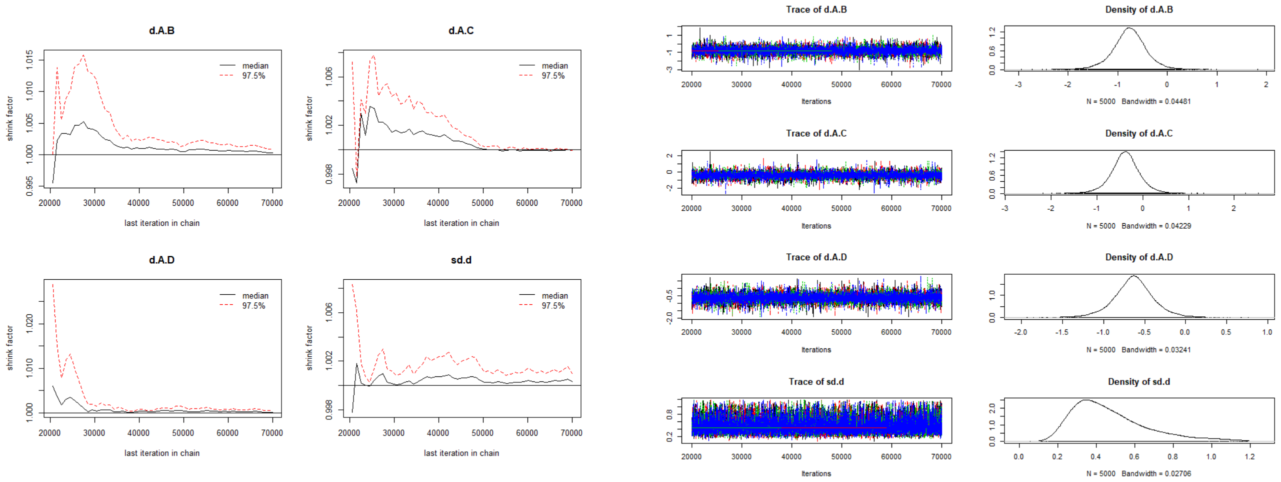

D. Intracranial hemorrhage

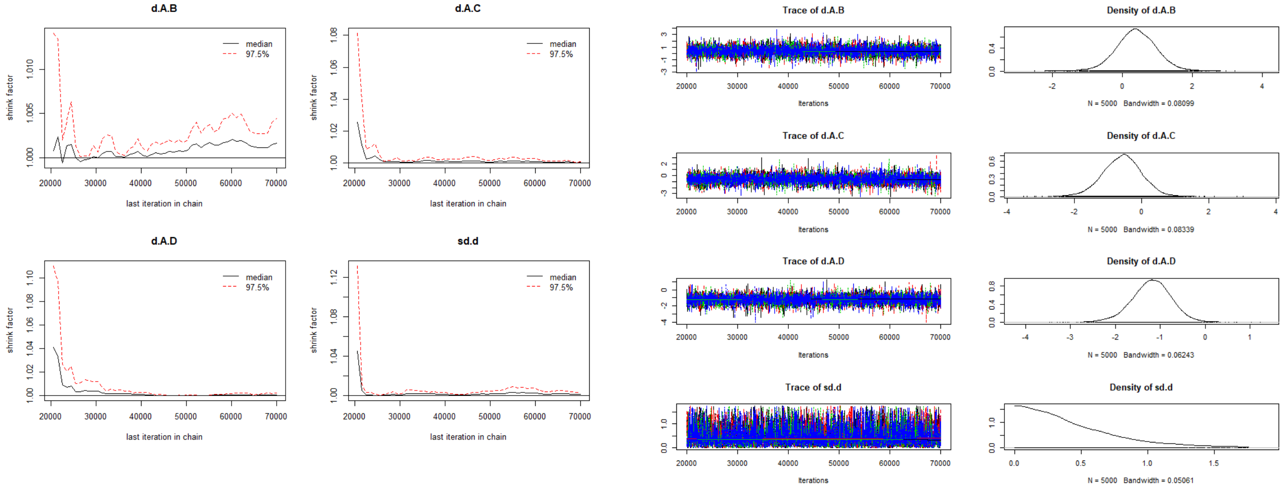

E. Trial-defined primary MACE outcome

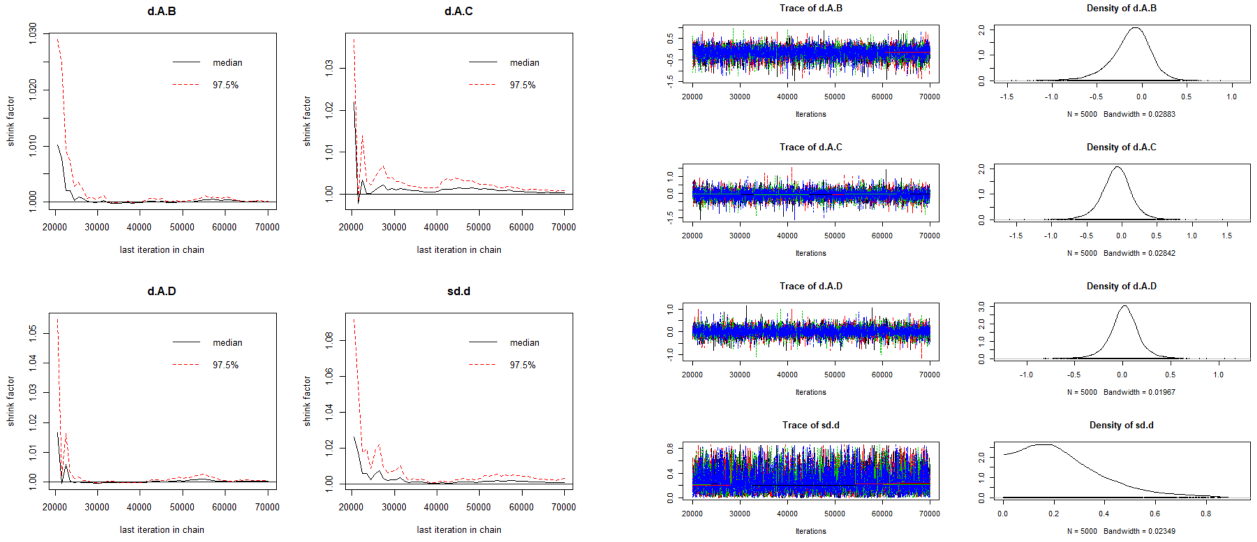

F. All cause death

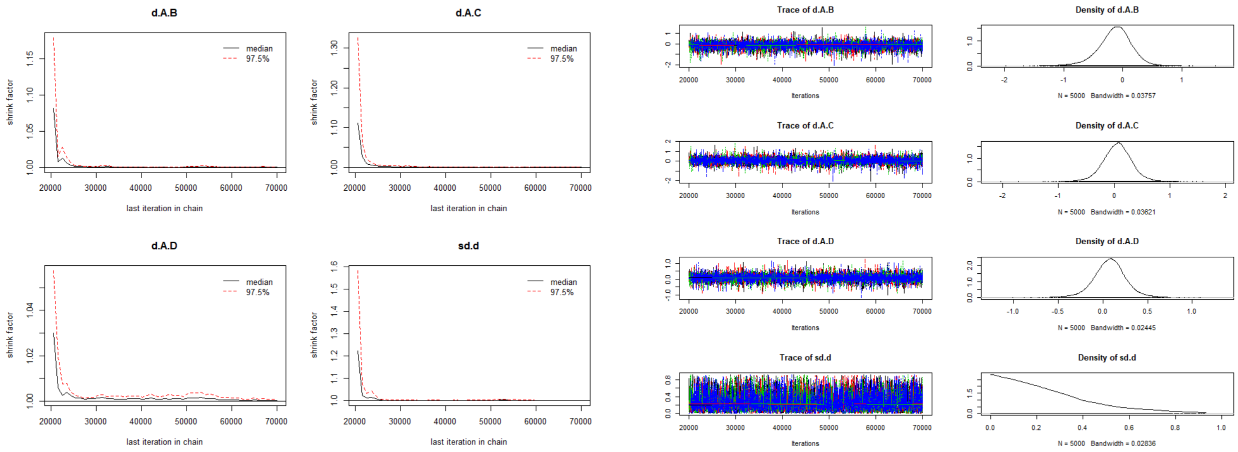

G. Cardiovascular death

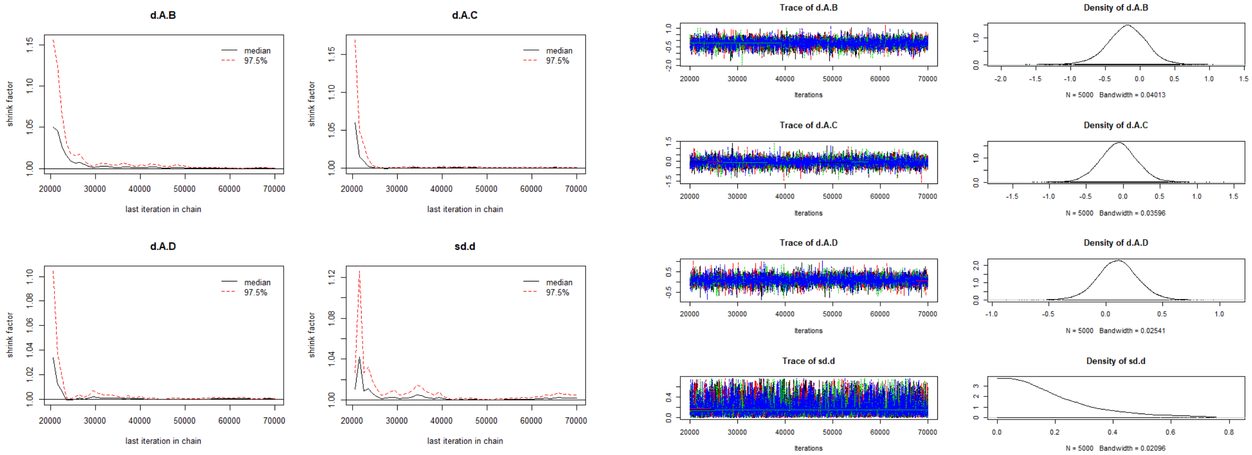

H. Myocardial infarction

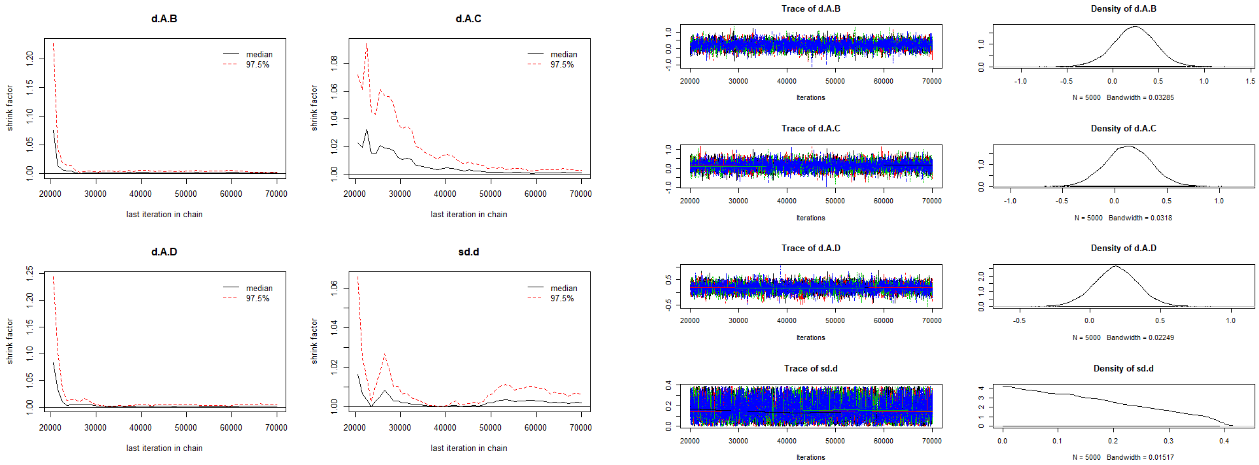

I. Stroke

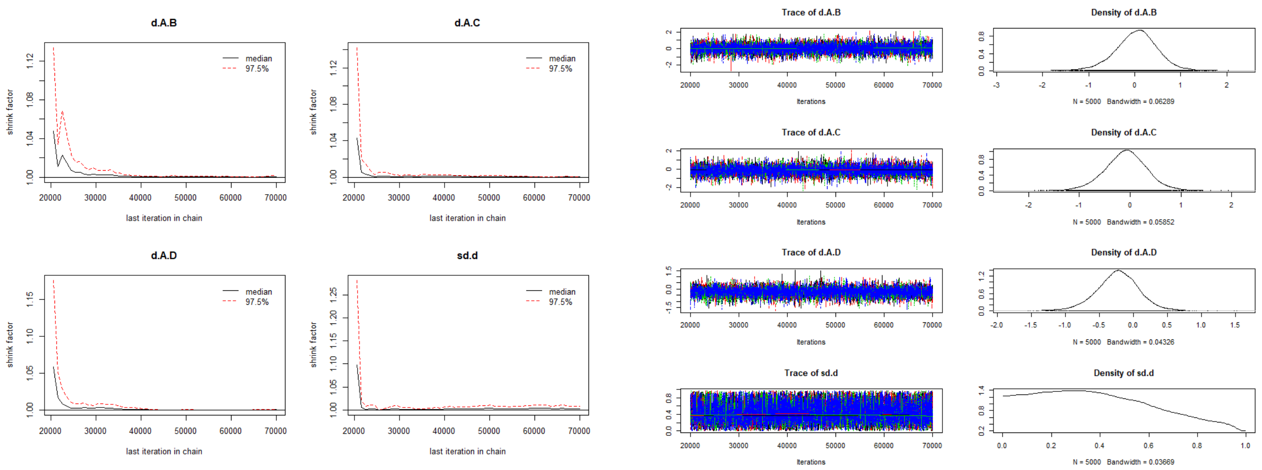

J. Stent thrombosis

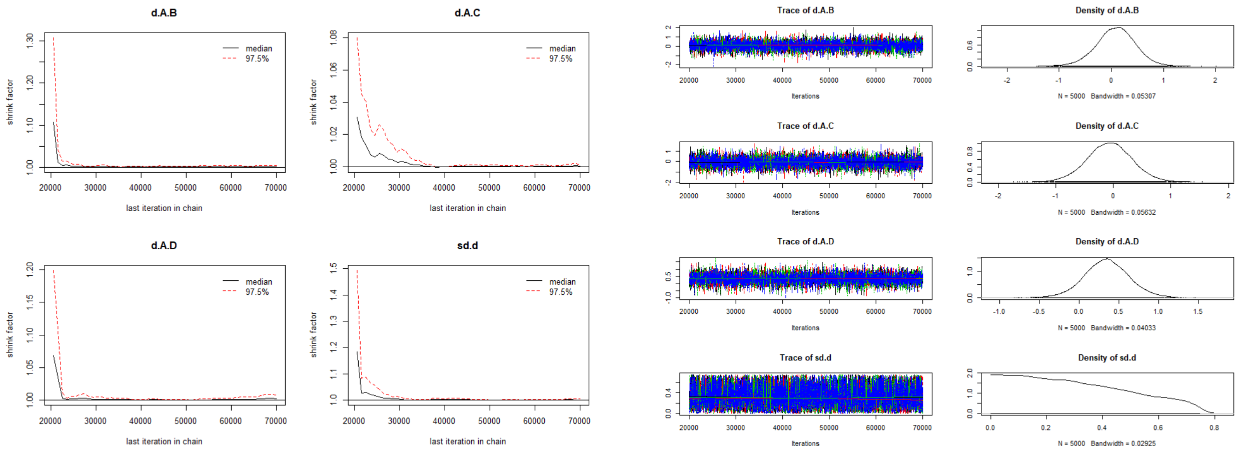

Supplementary Figure 2. Odds ratio (OR, 95% credible intervals, CrI) for safety outcomes among the four antithrombotic therapies. Odds ratio smaller than 1 means that the odds of having an event for the column treatment strategy is lower than the row treatment strategy. Statistical significance is indicated by bold, where the 95% CrI does not include 1.

A. TIMI major bleeding

**VKA with DAPT**	1.55 (0.92, 2.64)	1.37 (0.82, 2.38)	**1.90 (1.28, 2.83)**
0.64 (0.38, 1.09)	**VKA with SAPT**	0.89 (0.46, 1.72)	1.23 (0.68, 2.22)
0.73 (0.42, 1.22)	1.13 (0.58, 2.16)	**NOAC with DAPT**	1.39 (0.77, 2.41)
**0.53 (0.35, 0.78)**	0.82 (0.45,1.48)	0.720 (0.42,1.30)	**NOAC with $P2Y_{12}$ i**

B. TIMI major and minor bleeding

**VKA with DAPT**	1.73 (0.90, 3.20)	1.44 (0.72, 2.98)	**1.86 (1.09, 3.20)**
0.58 (0.31, 1.12)	**VKA with SAPT**	0.836 (0.38, 2.0)	1.08 (0.53, 2.35)
0.79 (0.34, 1.39)	1.20 (0.50, 2.66)	**NOAC with DAPT**	1.30 (0.62, 2.67)
**0.54 (0.31, 0.92)**	0.926 (0.43, 1.89)	0.771 (0.37, 1.63)	**NOAC with $P2Y_{12}$ i**

C. Trial-defined safety outcome

**VKA with DAPT**	**1.97 (1.13, 3.45)**	1.43 (0.77, 2.72)	**1.85 (1.16, 2.99)**
**0.51 (0.29, 0.89)**	**VKA with SAPT**	0.73 (0.35, 1.55)	0.94 (0.49, 1.81)
0.70 (0.37, 1.30)	1.38 (0.65, 2.89)	**NOAC with DAPT**	1.29 (0.68, 2.49)
**0.54 (0.33, 0.86)**	1.06 (0.55, 2.06)	0.78 (0.40, 1.47)	**NOAC with $P2Y_{12}$ i**

D. Intracranial hemorrhage

**VKA with DAPT**	0.67 (0.21, 2.25)	1.76 (0.56, 6.02)	**3.25 (1.41, 8.41)**
1.50 (0.44, 4.85)	**VKA with SAPT**	2.66 (0.63, 11.6)	**4.90 (1.29, 19.7)**
0.57 (0.17, 1.80)	0.38 (0.09, 1.60)	**NOAC with DAPT**	1.85 (0.489, 7.11)
**0.31 (0.12, 0.71)**	**0.20 (0.05, 0.77)**	0.540 (0.141, 2.04)	**NOAC with $P2Y_{12}$ i**

Supplementary Figure 3. Odds ratio (OR, 95% credible intervals, CrI) for efficacy outcomes among the four antithrombotic therapies. Odds ratio smaller than 1 means that the odds of having an event for the column treatment strategy is lower than the row treatment strategy. Statistical significance is indicated by bold, where the 95% CrI does not include 1.

A. Trial-defined MACE

**VKA with DAPT**	1.11 (0.77, 1.91)	1.08 (0.69, 1.74)	0.98 (0.70, 1.39)
0.90 (0.53, 1.30)	**VKA with SAPT**	0.969 (0.51, 1.57)	0.88 (0.49, 1.34)
0.93 (0.58, 1.44)	1.03 (0.64, 1.95)	**NOAC with DAPT**	0.91 (0.56, 1.43)
1.02 (0.72, 1.42)	1.13 (0.74, 2.03)	1.09 (0.70, 1.79)	**NOAC with $P2Y_{12}$ i**

B. All-cause death

**VKA with DAPT**	1.18 (0.75, 2.05)	0.95 (0.57, 1.62)	0.94 (0.65, 1.35)
0.85 (0.49, 1.33)	**VKA with SAPT**	0.81 (0.41, 1.43)	0.79 (0.43, 1.30)
1.05 (0.62, 1.76)	1.24 (0.70, 2.42)	**NOAC with DAPT**	0.98 (0.59, 1.65)
1.07 (0.74, 1.55)	1.27 (0.77, 2.33)	1.02 (0.61, 1.76)	**NOAC with $P2Y_{12}$ i**

C. Cardiovascular death

**VKA with DAPT**	1.37 (0.81, 2.50)	1.11 (0.66, 1.89)	0.922 (0.63, 1.35)
0.73 (0.40, 1.23)	**VKA with SAPT**	0.81 (0.41, 1.50)	0.675 (0.36, 1.17)
0.90 (0.53, 1.51)	1.23 (0.67, 2.46)	**NOAC with DAPT**	0.829 (0.48, 1.41)
1.08 (0.74, 1.59)	1.48 (0.86, 2.81)	1.21 (0.71, 2.07)	**NOAC with $P2Y_{12}$ i**

D. Myocardial infarction

**VKA with DAPT**	0.768 (0.47, 1.32)	0.86 (0.52, 1.43)	0.83 (0.57, 1.21)
1.30 (0.76, 2.14)	**VKA with SAPT**	1.12 (0.60, 2.0)	1.07 (0.60, 1.83)
1.16 (0.70, 1.93)	0.891 (0.50, 1.68)	**NOAC with DAPT**	0.95 (0.58, 1.61)
1.21 (0.83, 1.76)	0.933 (0.55, 1.65)	1.05 (0.62, 1.73)	**NOAC with $P2Y_{12}$ i**

E. Stroke

**VKA with DAPT**	0.95 (0.44, 2.24)	1.08 (0.48, 2.56)	1.25 (0.69, 2.39)
1.05 (0.45, 2.29)	**VKA with SAPT**	1.14 (0.41, 3.07)	1.32 (0.53, 3.25)
0.92 (0.39, 2.09)	0.88 (0.33, 2.44)	**NOAC with DAPT**	1.16 (0.48, 2.84)
0.80 (0.42, 1.45)	0.76 (0.31, 1.90)	0.86 (0.35, 2.07)	**NOAC with $P2Y_{12}$ i**

F. Stent thrombosis

**VKA with DAPT**	0.93 (0.45, 2.07)	1.08 (0.49, 2.41)	0.72 (0.41, 1.27)
1.07 (0.48, 2.21)	**VKA with SAPT**	1.17 (0.44, 2.80)	0.77 (0.33, 1.65)
0.93 (0.41, 2.05)	0.86 (0.36, 2.27)	**NOAC with DAPT**	0.66 (0.30, 1.48)
1.40 (0.79, 2.47)	1.30 (0.61, 3.03)	1.52 (0.68, 3.31)	**NOAC with $P2Y_{12}$ i**

Supplementary Table 1. Characteristics of the included trials

	WOEST	ISAR-TRIPLE*	PIONEER AF-PCI	RE-DUAL PCI	AUGUSTUS	ENTRUST-AF PCI
Target population	Patients taking VKA undergoing PCI	Patients taking VKA undergoing PCI	AF patients undergoing PCI	AF patients undergoing PCI	AF patients with recent ACS and/or PCI	AF patients undergoing PCI for SA or ACS
Timespan	Nov 2008–Nov 2011	Sept 2008–Dec 2013	May 2003–Jul, 2015	Jul 2014–Oct, 2016	Jun 2015–Apr, 2018	Feb 2017–Jun, 2019
Number of patients	573	614	2,124	2,725	4,614	1,508
Study type	Multicenter RCT	Multicenter RCT	Multicenter RCT	Multicenter RCT	2x2 factorial RCT	Multicenter RCT
Inclusion criteria	■ Age 18–80 y<br>■ Under VKA<br>■ CAD undergoing PCI	■ Age > 18 y<br>■ Under VKA<br>■ CAD undergoing PCI	■ Age ≥ 18 y<br>■ NVAF with PCI	■ Age ≥ 18 y<br>■ NVAF under PCI for ACS or stable CAD	■ Age ≥ 18 y<br>■ AF with recent ACS and/or PCI	■ Age ≥ 18 y<br>■ NVAF undergoing PCI for ACS or stable CAD
Exclusion criteria	■ History of ICH<br>■ Cardiogenic shock<br>■ Contraindication to aspirin, clopidogrel<br>■ Peptic ulcer in past 6 m<br>■ Thrombocytopenia<br>■ TIMI major in past 12 m	■ Previous stent thrombosis<br>■ DES in left main<br>■ Active bleeding<br>■ History of intracranial hemorrhage	■ History of stroke or TIA<br>■ Gastrointestinal bleeding within 12 m<br>■ CCr < 30 ml per min<br>Anemia (Hb<10 g/dL)	■ Presence of bioprosthetic or mechanical heart valves<br>■ CCr < 30 ml per min	■ Presence of prosthetic heart valves, venous thrombus, or mitral stenosis; coagulopathy<br>■ Severe renal insufficiency<br>■ History of ICH<br>■ Recent plan for CABG	■ Presence of mechanical heart valves<br>■ Moderate-to-severe mitral stenosis,<br>■ End-stage renal disease
Treatment strategies	■ VKA + P_2Y_{12} inhibitor<br>■ VKA + DAPT	■ VKA + P_2Y_{12} inhibitor<br>■ VKA + DAPT	■ R (15) + P_2Y_{12} inhibitor<br>■ R (2.5) + DAPT<br>■ VKA + DAPT	■ D (L) + P_2Y_{12} inhibitor<br>■ D (H) + P_2Y_{12} inhibitor<br>■ VKA + DAPT	■ A + P_2Y_{12} inhibitor<br>■ A + DAPT<br>■ VKA + P_2Y_{12} inhibitor<br>■ VKA + DAPT	■ E + P_2Y_{12} inhibitor<br>■ VKA + DAPT
$P2Y_{12}$ inhibitor	■ 100% clopidogrel	■ 100% clopidogrel	■ 94% Clopidogrel<br>■ 1% Prasugrel<br>■ 4% Ticagrelor	■ 86% Clopidogrel<br>■ 12% Ticagrelor	■ 93% Clopidogrel<br>■ 1% Prasugrel<br>■ 6% Ticagrelor	■ 92% Clopidogrel<br>■ 1% Prasugrel<br>■ 7% Ticagrelor
Primary safety outcome	Any bleeding classified by TIMI, GUSTO, and BARC	Any bleeding classified by TIMI, BARC	Bleeding according to TIMI	Major or CRNM bleeding according to ISTH	Major and CRNM bleeding according to ISTH	Any bleeding according to ISTH, BARC, or TIMI
Primary MACE outcome	Death, MI, stroke, TVR or ST	Death, MI, stroke, ST or major bleeding	Death, MI, stroke	Death, MI, stroke, SE and unplanned PCI	All-cause death or ischemic event	CV death, stroke, SE, MI, ST

*Only data of landmark analysis from 6 weeks to 9 months was included in the present study

A: Apixaban; ACS: Acute coronary syndrome; AF: Atrial fibrillation; BARC: Bleeding Academic Research Consortium; CABG: Coronary artery bypass grafting; CAD: Coronary artery disease; CCr: Clearance of creatinine; CRNM bleeding: Clinically relevant non-major bleeding; CV death: Cardiovascular death; D (H): Dabigatran 150 mg twice daily; D (L): Dabigatran 110 mg twice daily; DAPT: Dual antiplatelet therapy; DES: Drug eluting stent; E: Edoxaban; GUSTO: Global Utilization of Streptokinase and Tissue Plasminogen Activator for Occluded Coronary Arteries; ICH: Intracranial hemorrhage; ISTH: International Society on Thrombosis and Hemostasis; TIMI: Thrombolysis in myocardial infarction; MACE: Major adverse cardiovascular events; MI: Myocardial infarction; NVAF: Non-valvular atrial fibrillation; PCI: Percutaneous coronary intervention; R (15): Rivaroxaban 15 mg once daily; R (2.5): Rivaroxaban 2.5 mg twice daily; RCT: Randomized controlled trial; SE: Systemic embolism; ST: stent thrombosis; TIA: Transient ischemic attack; TVR: Target vessel revascularization; VKA: Vitamin K antagonist

Supplementary Table 2. Safety and Efficacy outcome of the included trials

	WOEST		ISAR-TRIPLE*		PIONEER AF-PCI			RE-DUAL PCI		AUGUSTUS				ENTRUST-AF PCI	
Antithrombotic strategies	VKA + DAPT	VKA + SAPT	VKA + DAPT	VKA + SAPT	VKA + DAPT	NOAC + DAPT	NOAC + $P2Y_{12}i$	VKA + DAPT	NOAC + $P2Y_{12}i$	VKA + DAPT	VKA + SAPT	NOAC + DAPT	NOAC + $P2Y_{12}i$	VKA + DAPT	NOAC + $P2Y_{12}i$
■ Safety outcome (N)	284	279	307	307	697	706	696	981	1744	1123	1126	1145	1143	755	751
TIMI major bleeding	16	9	7	7	20	12	14	37	30	29	18	25	13	24	15
TIMI major and minor bleeding	89	39	15	14	33	19	21	69	56	80	51	64	32	144	124
Trial-defined primary safety outcome	126	54	70	48	167	117	109	264	305	208	122	157	84	152	128
Intracranial hemorrhage	3	3	0	0	7	3	3	10	4	4	8	4	1	9	4
■ Efficacy outcome (N)	284	279	307	307	695	704	694	981	1744	1154	1154	1153	1153	755	751
Trial-defined primary MACEs	50	31	10	4	36	36	41	131	239	66	84	71	72	46	49
All-cause death	18	7	12	8	13	17	16	48	85	34	40	38	39	37	46
Cardiovascular death	7	3	8	3	11	14	15	31	58	28	26	25	32	16	17
Myocardial infarction	13	9	0	1	14	21	17	29	70	34	46	34	38	23	29
Stroke	8	3	2	2	7	10	8	13	26	12	14	8	5	12	10
Stent thrombosis	9	4	0	0	4	6	5	8	22	12	19	11	21	6	8

Values are presented as patient or event number of each trial.

*Only data of landmark analysis from 6 weeks to 9 months was included in the present study.

DAPT: Dual antiplatelet therapy; MACE: Major adverse cardiovascular event; NOAC: non-vitamin K oral anticoagulant; $P2Y_{12}i$: $P2Y_{12}$ inhibitor; SAPT: Single antiplatelet therapy; TIMI: Thrombolysis in myocardial infarction.
